# Physical ExeRcise Following Esophageal Cancer Treatment (PERFECT) study: design of a randomized controlled trial

**DOI:** 10.1186/s12885-017-3542-8

**Published:** 2017-08-18

**Authors:** Jonna K. van Vulpen, Peter D. Siersema, Richard van Hillegersberg, Grard A. P. Nieuwenhuijzen, Ewout A. Kouwenhoven, Richard P. R. Groenendijk, Donald L. van der Peet, Eric J. Hazebroek, Camiel Rosman, Carlo C. G. Schippers, Elles Steenhagen, Petra H. M. Peeters, Anne M. May

**Affiliations:** 1 Julius Center for Health Sciences and Primary Care, University Medical Center Utrecht, P.O. Box 85500, STR 6.131, 3508 GA Utrecht, The Netherlands; 2 Department of Gastroenterology and Hepatology, University Medical Center Utrecht, Heidelberglaan 100, 3584 CG Utrecht, The Netherlands; 3 Department of Gastroenterology and Hepatology, Radboud University Medical Center, P.O. Box 9101, 6500 HB Nijmegen, The Netherlands; 4 Department of Surgery, University Medical Center Utrecht, Heidelberglaan 100, 3584 CG Utrecht, The Netherlands; 5 Department of Surgery, Catharina Hospital, Michelangelolaan 2, 5623 EJ Eindhoven, The Netherlands; 6 Department of Surgery, ZGT Hospital, Zilvermeeuw 1, 7609 PP Almelo, The Netherlands; 7 Department of Surgery, IJsselland Hospital, Prins Constantijnweg 2, 2906 ZC Capelle a/d IJssel, The Netherlands; 8 Department of Surgery, VU University Medical Center, De Boelelaan 1117, 1081 HV Amsterdam, The Netherlands; 9 Department of Surgery, St. Antonius Hospital, Koekoekslaan 1, 3430 VB Nieuwegein, The Netherlands; 10 Department of Surgery, Radboud University Medical Center, P.O. Box 9101, 6500 HB Nijmegen, The Netherlands; 11 Department of Dietetics, University Medical Center Utrecht, Heidelberglaan 100, 3584 CG Utrecht, The Netherlands; 12 School of Public Health, Imperial College London, South Kensington Campus, London, SW7 2AZ UK

**Keywords:** Esophageal cancer, Physical exercise, Quality of life, Randomized controlled trial

## Abstract

**Background:**

Following esophagectomy, esophageal cancer patients experience a clinically relevant deterioration of health-related quality of life, both on the short- and long-term. With the currently growing number of esophageal cancer survivors, the burden of disease- and treatment-related complaints and symptoms becomes more relevant. This emphasizes the need for interventions aimed at improving quality of life. Beneficial effects of post-operative physical exercise have been reported in several cancer types, but so far comparable evidence in esophageal cancer patients is lacking. The aim of this study is to investigate effects of physical exercise on health-related quality of life in esophageal cancer patients following surgery.

**Methods:**

The Physical ExeRcise Following Esophageal Cancer Treatment (PERFECT) study is a multicenter randomized controlled trial including 150 esophageal cancer patients after surgery with curative intent. Patients are randomly allocated to an exercise group or usual care group. The exercise group participates in a 12-week combined aerobic and resistance exercise program, supervised by a physiotherapist near the patient’s home-address. In addition, participants in the exercise group are requested to be physically active for at least 30 min per day, every day of the week. Participants allocated to the usual care group are asked to maintain their habitual physical activity pattern. The primary outcome is health-related quality of life (EORTC-QLQ-C30). Secondary outcomes include esophageal cancer specific quality of life, fatigue, anxiety and depression, sleep quality, work-related factors, cardiorespiratory fitness (VO_2peak_), muscle strength, physical activity, malnutrition risk, anthropometry, blood markers, recurrence of disease and survival. All questionnaire outcomes, diaries and accelerometers are assessed at baseline, post-intervention (12 weeks post-baseline) and 24 weeks post-baseline. Physical fitness, anthropometry and blood markers are assessed at baseline and post-intervention. In addition, adherence and safety are monitored throughout the exercise program.

**Discussion:**

This randomized controlled trial investigates effects of physical exercise versus usual care in esophageal cancer patients after surgery. As the design of the exercise program closely resembles daily practice, this study can contribute both to evidence on effects of exercise in esophageal cancer patients, and to potential implementation strategies.

**Trial registration:**

Trial registration:Netherlands Trial Registry NTR5045

Date of trial registration: January 19th, 2015

Date and version study protocol: February 2017, version 1

## Background

Age standardized (world population) incidence rates for esophageal cancer have shown a rather steep upward trend in the last decades in the Netherlands (from 3.73/100,000 in 1990 to 6.89/100,000 in 2015) [1]. Furthermore, survival of esophageal cancer has slowly improved over the past 25 years, as a result of improvements in diagnosis and treatment of the disease [1, 2]. In the 30–40% of patients who are eligible for a potential curative treatment, the combination of surgery with neoadjuvant chemoradiotherapy has provided a considerable survival benefit compared to surgery alone (Hazard Ratio: 0.78; 95% Confidence Interval (CI) 0.70 to 0.88) [3], leading to a 5-year survival of 47% in this particular group of patients [4].

On the downside, a clinically relevant deterioration of health-related quality of life (HRQoL) is observed both during preoperative chemoradiotherapy and after esophagectomy [5, 6]. After surgery, esophageal cancer patients experience several disease- and treatment-related problems, such as fatigue, eating difficulties, diarrhoea, nausea and vomiting, both on the short- and long-term [7, 8]. In general, impaired HRQoL outcomes last for 9–12 months, although some can even persist beyond the first year after surgery [6, 9]. With a growing number of esophageal cancer survivors, the burden of disease- and treatment-related problems increases, and interventions aimed at improving HRQoL are needed.

Several meta-analyses have shown beneficial effects of physical exercise interventions after completion of treatment on HRQoL [10–12]. Included randomized controlled trials (RCTs) have been performed in various cancer types, such as colorectal carcinoma, lymphoma and, mostly, breast cancer. Since patient and treatment characteristics of esophageal cancer patients are different, results from these RCTs and meta-analyses may not be directly generalizable to this patient population.

So far, no RCT has been performed to investigate whether beneficial effects of physical exercise interventions also apply for esophageal cancer patients. Therefore, the Physical ExeRcise Following Esophageal Cancer Treatment (PERFECT) study was initiated, to investigate the effects of a 12-week combined aerobic and resistance exercise program in esophageal cancer patients after surgery with curative intent on health-related quality of life. In this manuscript, we report the design of the PERFECT study.

## Methods

### Design

The primary aim of the PERFECT study is to investigate effects of physical exercise on health-related quality of life in esophageal cancer patients after surgery with curative intent. In addition, we study the effects on fatigue, physical fitness, physical activity, functional wellbeing and symptoms, esophagus-specific QoL, anxiety and depression, diet, sleep quality, anthropometry, work-related factors and blood markers (secondary outcomes). Furthermore, we will explore the effect of physical exercise on disease recurrence and overall survival 5 years after diagnosis. The PERFECT study is designed as a multicenter, randomized controlled trial with two study arms: (1) a group offered a supervised exercise program in addition to usual care and (2) a control group receiving usual care without exercise intervention. We hypothesize that physical exercise leads to higher levels of quality of life, compared with usual care. The PERFECT study protocol has been approved by the Medical Ethics Committee of the University Medical Center Utrecht in December 2014. The study is registered with the Netherlands Trial Registry under NTR5045.

### Participants

A total of 150 patients with esophageal cancer after surgery with curative intent are planned to be included in the PERFECT study. In order to be eligible for our study, a participant must meet all of the following inclusion criteria: surgery with curative intent for newly diagnosed, histologically confirmed esophageal cancer; 4 weeks to 1 year after hospital discharge following surgery; age ≥ 18 years; able to read and understand the Dutch language; physically inactive (≤150 min per week of moderate-vigorous exercise); Karnofsky Perfomance Status ≥60; able to walk ≥60 m. Exclusion criteria are: presence of metastatic esophageal cancer; irradical resection; contra-indications for physical activity (as assessed through the Revised Physical Activity Readiness Questionnaire [13]). Patients who are already involved in a supervised exercise program are also excluded. Written informed consent is obtained from all participants prior to participation in the study.

Depending on patient characteristics, surgical experience and preference, esophagectomy can be performed through transthoracic or transhiatal approach. Both approaches, as well as the use of either open or minimally invasive techniques for surgical resection, are allowed for inclusion in the PERFECT study. Both patients with and without neoadjuvant chemotherapy and/or radiotherapy treatment are eligible for the study.

### Recruitment and randomization

**Fig. 1:**
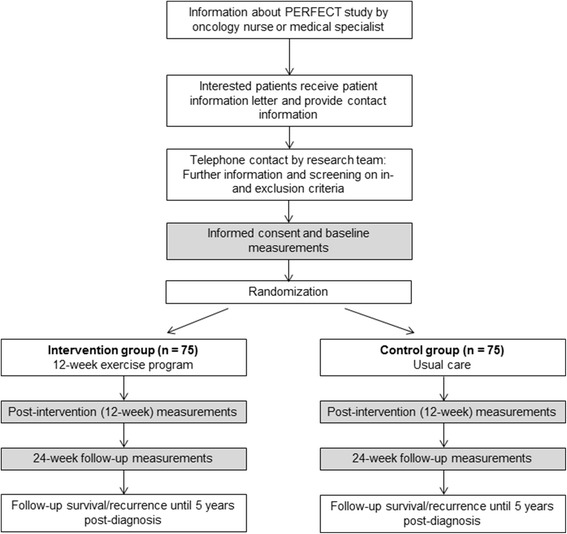
Recruitment and allocation procedure

Participants are recruited from seven Dutch hospitals, specialized in esophageal cancer treatment. Dependent on recruitment rate, more hospitals might be invited to collaborate. The recruitment and allocation procedure is summarized in Fig. 1. Potentially eligible patients are informed about the study by the oncology nurse or medical specialist of each participating hospital. Interested patients provide their contact information and receive a patient information letter explaining the study aims and procedures. After 1 week, the investigator or research nurse contacts the patients by telephone to provide further information, answer remaining questions, and verify inclusion and exclusion criteria. Eligible patients who are willing to participate are invited to the study center to sign written informed consent and for baseline measurements. When participants have completed the baseline measurements, they are randomly allocated (1:1) to either the exercise intervention or usual care group, stratified by sex, hospital and time since surgery. Randomization is performed by central data management, using minimization. Allocation to the exercise intervention or usual care group is concealed. Due to the nature of the intervention, blinding of the participants towards allocation is not possible.

After randomization, patients randomized to the intervention group are informed on the supervised exercise program and follow-up measurements. Those randomized to the control group are informed on the continuation of usual care and the follow-up measurements. The total study duration for every participant is about 24 weeks. Participants who choose not to participate in the PERFECT study are asked to provide their reason for non-participation.

### Intervention

The standardized physical exercise program combines aerobic and resistance training and takes place twice weekly during 12 weeks. The individual components of the exercise program are described below in the sections ‘Aerobic training’ and ‘Resistance training’. All exercise sessions are supervised and guided by a physiotherapist, preferably an oncology physiotherapist. Since esophageal cancer care is centralized in the Netherlands, training at the treating hospitals might not be feasible for some patients due to relatively long travel distances. Before start of the PERFECT study, we interviewed 4 esophageal cancer patients, who all indicated a maximal travel distance between 5 and 15 km as acceptable. Therefore, the exercise program is offered close to home, either in a general physiotherapist practice or in a hospital nearby the patient’s home address. Each physiotherapist is trained by the PERFECT study team to perform the exercise sessions in a standardized way.

Although there is not yet enough evidence to draw definite conclusions about the most effective design of exercise programs [14], programs with moderate-to-vigorous intensity have been shown to have significant beneficial effects on HRQoL, whereas programs with mild-to-moderate intensity had not [11, 15]. With regard to the mode of physical activity, the ACSM guidelines on exercise for cancer survivors have included both aerobic exercise and resistance exercise [16, 17]. Therefore, we study the effectiveness of a moderate-to-vigorous intensity program, combining both aerobic and resistance exercise.

The exercise protocol has previously been shown to be safe and feasible in healthy overweight, postmenopausal women [18]. Exercise protocols with comparable content and intensity have been shown to be safe and feasible in patients with breast, colon, ovarian, cervix or testis cancer, or lymphomas [15, 19, 20]. The duration of every exercise session is 1 hour and includes a warming up (5 min), aerobic and resistance training (50 min) and a cooling down (5 min). In case of physical limitations, an adapted exercise program will be provided.

#### Aerobic training

The exercise protocol is individualized to the patient’s fitness level using heart rate reserve (HRR) as determined at baseline with cardiopulmonary exercise testing (CPET) for the aerobic training. The HRR represents the change between heart rate in rest and peak heart rate. Both parameters are tested at baseline with CPET, performed at the sports medicine or lung function department of the study center (see section ‘Study outcomes’). The physiotherapist who supervises the exercise program of the patient is informed about the HRR by the PERFECT study team.

Levels of exercise intensity gradually increase during the exercise program. During the first 3 weeks of the training, the endurance training consists of 15–20 min training on an exercise machine, e.g. a treadmill, exercise bicycle or cross-trainer, at 40–60% of the HRR. From the fourth until the eighth week of the training, participants train 15–20 min at 60–70% of the HRR and 5–10 min at 70–89% HRR. From the ninth week onwards, participants train 10 min at 60–75% HRR, and in addition they perform interval training: 10 × 30 s vigorous to maximal exercise, alternated with a 1-min active rest (Table 1). Participants wear heart rate monitors to ensure exercise training at the prescribed intensity.

**Table 1:** Exercise protocol

Week	Aerobic training	Resistance training
1–3	15–20 min 40–60% HRR	One set of 20–25 repetitions at 20-RM weight for each exercise^a^
4–8	15–20 min 60–70% HRR + 5–10 min 70–89% HRR	
9–12	10 min 60–75% HRR + Interval training: 10 × 30 s vigorous to maximal exercise, alternated with a 1-min active rest.	Two sets of 15–20 repetitions at 15-RM weight for each exercise^b^

^a^Rowing, bench press, squat, shoulder press, biceps curl, lunges, calf-raises, triceps extension, abdominal crunch

^b^Rowing, bench press, squat, shoulder press, biceps curl, triceps extension, abdominal crunch/hoover

#### Resistance training

For resistance training, the exercise protocol is also individualized to the patient’s fitness level using 20-repetition maximum (20-RM) muscle strength tests as determined at the intake session by the physiotherapist. Intensities increase from one set of 20–25 repetitions at the 20-RM weight to a maximum of two sets of 15–20 repetitions at 15-RM weight (Table 1). To secure sufficient training load throughout the training program, the 20-RM muscle strength test will be repeated at week 4, and a 15-RM test will be determined at week 8, with the resistance being adjusted accordingly. Resistance training is performed for the major muscle groups and consists of the following exercises: rowing, bench press, squat, shoulder press, biceps curl, lunges, calf-raises, triceps extension and abdominal crunch/hoover.

#### Home-based exercise

In addition to the supervised exercise program, participants are asked to be physically active for at least 30 min a day on all remaining days of the week, according to the WCRF/AICR guidelines for cancer survivors [21]. This should include an aerobic component of moderate intensity in agreement with the participant’s fitness and preferences. During an intake session, patients are supported by their physiotherapist to set appropriate exercise goals. Participants document their activities daily in an exercise log. Every 2 weeks, the exercise log is discussed with the physiotherapist, to evaluate progress of physical activities and expand existing exercise goals and/or set new exercise goals.

### Usual care group

Participants in the usual care group receive usual care and are requested to maintain their habitual physical activity pattern.

### Study outcomes

Table 2 summarizes the outcomes measured in the PERFECT study. All questionnaire outcomes, diaries and the accelerometers are assessed at baseline, post-intervention (12 weeks post-baseline) and 24 weeks post-baseline. At baseline and post-intervention these outcomes are assessed during a study center visit. At 24-weeks participants receive the questionnaires by mail. Physical fitness and blood markers are assessed at baseline and post-intervention. Recurrence and survival will be assessed 2- and 5-year post-diagnosis. Socio-demographic data (age, sex, education, marital status) and smoking status are assessed at baseline with a self-developed questionnaire. Medical data (diagnosis, tumor type, disease stage, type of treatment, and comorbidities) are retrieved from medical records. Personal data are coded and all data are handled by the researchers in compliance with the Dutch Personal Data Protection Act. The PERFECT study is checked on safety for participants and validity of data by an independent monitor.

**Table 2:** Study outcomes

Outcomes	Instrument	Baseline	12-week	24-week
Primary outcome				
Quality of life	EORTC-QLQ-C30	X	X	X
Secondary outcomes				
Esophageal cancer specific symptoms	EORTC-QLQ-OG25	X	X	X
Fatigue	Multidimensional Fatigue Inventory (MFI)	X	X	X
Anxiety and depression	Hospital Anxiety and Depression Scale (HADS)	X	X	X
Sleep quality	Pittsburgh Sleep Quality Index (PSQI)	X	X	X
Work-related factors	iMTA Productivity Cost Questionnaire (iPCQ)	X	X	X
Cardiorespiratory fitness	Cardiopulmonary exercise testing (CPET)	X	X	
Muscle strength	MicroFET handheld dynamometer	X	X	
Physical activity	Short questionnaire to assess health enhancing physical activity (SQUASH)	X	X	X
	ActiGraph accelerometer	X	X	X
	Exercise log		X	
Malnutrition risk	Patient-Generated Subjective Global Assessment Short Form (PG-SGA SF)	X	X	X
Dietary intake	3-Day food diary	X	X	X
Anthropometry	Body weight, height, BMI, waist and hip circumference	X	X	
Blood parameters	Serum, plasma and cell pellet	X	X	
Adherence and compliance	Registration in case report form, exercise log	X	X	
Recurrence and survival	Medical records and Dutch Cancer Registry			
Other				
Sociodemographic data	Self-developed questionnaire	X		
Medical data	Medical records	X		
Adverse events	Reports of patients, physiotherapists, oncology nurses, physicians, medical records	X	X	X

#### Quality of life

To measure HRQoL, the study’s primary outcome, the global quality of life subscale of the validated, 30-item European Organisation Research and Treatment of Cancer-Quality of Life-C30 questionnaire (EORTC-QLQ-C30; version 3) is used. For this study, the Dutch translation of the EORTC-QLQ-C30 is used. In addition to the quality of life subscale, the EORTC-QLQ-C30 incorporates five functional subscales (physical, role, emotional, cognitive and social), three symptom scales (fatigue, nausea & vomiting, and pain) and six single items (dyspnea, insomnia, appetite loss, constipation, diarrhea, and financial difficulties). All scale and item scores range from 0 to 100. Higher scores on the global quality of life subscale indicate a higher QoL. In the functional subscales, a higher score is equivalent to better levels of function, whereas in the symptom scales and single items a higher score is indicative of more symptoms [22].

Esophageal cancer specific problems are assessed with the validated 25-item oesophagogastric module (QLQ-OG25). This module consists of six symptom scales: dysphagia, eating restrictions, reflux, odynophagia, pain, and anxiety. Scores range from 0 to 100, with higher scores representing more symptoms [23].

#### Fatigue

Fatigue is measured using the validated, Dutch version of the Multidimensional Fatigue Inventory (MFI). The MFI is a 20-item questionnaire, designed to measure the following dimensions: general fatigue, physical fatigue, mental fatigue, reduced activity and reduced motivation. Scores range from 0 to 20, with higher scores indicating more fatigue [24].

#### Anxiety and depression, sleep quality and work-related factors

Anxiety and depression are assessed using the Dutch language version of the self-report Hospital Anxiety and Depression Scale (HADS). The HADS consists of two subscales, the depression subscale and anxiety subscale. Both subscales have a score range from 0 to 21, with higher scores indicating more depression or anxiety [25, 26]. Sleep quality is assessed using the Pittsburgh Sleep Quality Index (PSQI), which is a self-administered questionnaire including four open-ended questions and 14 questions based on a scale assessing subjective sleep quality, sleep latency, sleep duration, habitual sleep efficiency, sleep disturbances, sleep-promoting medication use, and daytime dysfunction over the previous 1 month [27]. Work-related factors are measured using the iMTA Productivity Cost Questionnaire (iPCQ), which is developed as a self-report instrument to measure and value productivity losses, incorporating three modules: productivity losses of paid work due to absenteeism, productivity losses of paid work due to presenteeism, and productivity losses related to unpaid work [28].

#### Physical fitness

Physical fitness is assessed as cardiorespiratory fitness and muscle strength. Cardiorespiratory fitness is determined using symptom-limited CPET with continuous breathing gas analysis on a bicycle ergometer, and is performed under medical supervision. CPET is proven to be safe for cancer patients prior to a physical exercise program [29]. After a 1-min warm-up under no-load, cycling workload is gradually increased with a predetermined 10, 15 or 20 W per minute, depending on the patient’s condition. Patients are instructed to cycle with a frequency of 70–80 rpm (revolutions per minute), until exhaustion sets in. The test is terminated when cycling frequency drops below 70 RPM or by decision of the physician, and is followed by a 3-min cooling-down at 20 W. During the test, information is provided through continuous 12-lead electrocardiography, blood pressure monitoring and breath-by-breath analysis. Peak oxygen uptake (VO_2peak_) is determined by taking the mean of VO_2_ values of the last 30 s before exhaustion. In addition, peak work load, peak heart rate and VO_2_ at ventilatory threshold are assessed. In this study, performance of CPET serves three goals: 1) measuring cardiorespiratory fitness, 2) medical evaluation and exercise clearance, and 3) determining the HRR at baseline to estimate individual training intensity for patients participating in the exercise program.

Muscle strength of quadriceps is measured with a microFET2® hand held dynamometer, using the ‘break technique’. Hereby, the patient is seated on an examination table with his/her knees in 90^0^ flexion. The patient is instructed to gradually increase knee extension force to a maximum, and sustain for 3 s. The examiner then overpowers this maximum, thereby providing a measurement of eccentric force. The procedure is repeated three times, for the left and right leg alternately, while the patient is verbally encouraged. In case of a difference of >10% between the two highest measurements, a fourth measurement is taken.

#### Physical activity

Physical activity behavior is assessed using the short questionnaire to assess health enhancing physical activity (SQUASH), which includes items on commuting activities, leisure time activities, household activities, and activities at work and school [30].

To assess physical activities (duration/intensity) and duration of sedentary behavior objectively, all participants wear an accelerometer (GT3X+ Tri-Axis Actigraphy Monitor, ActiGraph®) for seven consecutive days at baseline and follow-up measurements. In addition, participants in the intervention group are asked to keep an exercise log during the exercise program. In this exercise log, they document their daily activities.

#### Nutritional status and dietary intake

Malnutrition risk is detected at baseline and follow-up using the Patient-Generated Subjective Global Assessment Short Form (PG-SGA SF) [31, 32]. Furthermore, dietary intake at baseline and follow-up measurements is assessed using a 3-day food diary, which is filled out on two non-consecutive week days and one weekend day. Filled-in diaries are checked by a researcher or dietitian and are, in case of incompleteness, complemented during a telephone call with the participant. The food records will be coded and analyzed using the Dutch Food Composition Database (Nevo 2013, National Institute for Public Health and the Environment (RIVM), The Netherlands).

#### Anthropometry

Before the CPET, body weight and height are measured of participants wearing light clothes and no shoes. To measure body weight, we use a calibrated analogue balance and digital balance, depending on study center. Analogue values are rounded to the nearest 0.5 kg. Height is measured using a wall mounted tape measure, with values rounded to the nearest 0.5 cm. The same balance and tape measure are always used for all measurements of an individual participant. Body Mass Index (BMI) is calculated as weight in kilograms divided by height in meters squared (kg∙m^−2^). Waist and hip circumference (to the nearest 0.5 cm) are measured standing at the midway between lower ribs and iliac crest, and as the largest circumference between waist and thigh, respectively. Measurements are taken in duplicate and averaged.

#### Blood collection

Serum, plasma and cell pellet are derived from whole blood samples, processed within 4 hours and stored at -80 °C in the biobank of the University Medical Center Utrecht to be used for future analyses of biomarkers.

#### Adherence

Adherence incorporates both attendance at the supervised exercise sessions and compliance (i.e., performing the exercises according to exercise protocol). To monitor attendance, the physiotherapist documents presence of each participant at each session in a case report form. Furthermore, to monitor compliance, achieved heart rates and duration for the aerobic exercise components are documented, and weight and number of repetitions performed for the resistance components. In case of missed exercise sessions or non-compliance to the protocol, reasons are documented. At least every 4 weeks, this documentation is sent to the researchers for monitoring. Moreover, each physiotherapist is visited at least once by a member of the PERFECT study team, to ensure proper performance of the exercise protocol.

#### Recurrence and survival

Information on cancer recurrence, new primary tumors and (all-cause) death is retrieved from medical records and the Dutch Cancer Registry.

### Safety

CPET with electrocardiography is performed for exercise clearance before start of the exercise training. Adverse events reported by the patient or observed by physiotherapists, oncology nurses or physicians are recorded and serious adverse events are reported to the medical ethical committee. Malnutrition, dysphagia and weight loss are common problems in esophageal cancer patients [7], hence weight loss is not a goal of the exercise program. Body weight is therefore closely monitored by weekly supervised weighing by the physiotherapist. All physiotherapists are instructed to contact the researchers if weight loss exceeds 5% in 1 month, or 10% in total, upon which the study team will consult a dietitian or medical specialist.

### Sample size

The sample size is based on the primary outcome; improvement in HRQoL from baseline to post-intervention, assessed by the EORTC-QLQ-30. In our previous trial [33, 34], investigating the effects of a 12-week exercise training in cancer survivors, HRQoL in the intervention group improved by 15.1 (SD 17.7) points and the control group by 6.1 (SD 17.1) points; this difference was significant and clinically relevant [35]. We performed a power analysis with PASS, using these results and assuming a power of 80% (alpha = 0.05), which showed that 60 patients are needed in each group to detect an intervention effect. To take into account the correlation between baseline and follow-up in the sample size calculation, Borm et al. developed a method to calculate the sample size, in which the previously calculated number of subjects, should be multiplied by (1-ρ2), plus one extra subject per group [36]. ρ represents the correlation between baseline and follow-up outcomes. In our previous trials we found a correlation of 0.4 between baseline and follow-up HRQoL. By applying the method of Borm et al., we have calculated a sample size of 51 patients per group. We intend to include 75 patients per group taking a drop-out rate of approximately 30% into account (primarily due to early death). This sample size also enables us to detect intervention effects on the secondary outcomes (e.g. physical functioning, VO_2peak_ and fatigue). If drop-out appears to be lower, less patients per group can be included (e.g. in case of a drop-out rate of 10%, 57 patients per group can be included).

### Statistical analysis

Descriptive statistics will be used to describe the study population and study parameters at baseline. Questionnaire scores (e.g. HRQoL and fatigue) will be calculated according to published scoring algorithms. Analyses will be performed according to the intention-to-treat principle. For the primary analysis mixed linear regression models will be used to model the HRQoL outcome measures at 12 and 24 weeks. The models will be adjusted for baseline HRQoL as well as for stratification factors. In these longitudinal analyses, the program accounts for missing data based on the observed data [37]. The same analyses will be performed for the secondary outcome measures. Secondary outcomes, which will only be assessed at baseline and 12 weeks (e.g. VO_2peak_), will be analyzed as between-group differences in outcomes using ANCOVA, adjusted for baseline and stratification factors. Cancer recurrence and survival will be analyzed using a Cox proportional hazards regression model. As an explorative analysis, attendance and compliance, sex, subtype of carcinoma, type of surgery and time since surgery will be examined as potential modifiers of the intervention effects. Furthermore, to adjust for treatment contamination, an instrumental variable analysis will be performed with treatment assignment as instrument and adjustment of the results for adherence [38].

## Discussion

The aim of the PERFECT study is to investigate effects of exercise on HRQoL in esophageal cancer patients after surgery. Results of exercise on HRQoL in other types of cancer are promising, however effects in esophageal cancer patients have not been assessed before.

A special feature of the current study is the supervision of the exercise program by physiotherapists near the home-address of the participants who are randomized to the intervention group. As this approach diminishes travel burden considerably, we expect it to improve adherence of the participants. It might be challenging though, as for each individual participant an appropriate physiotherapist needs to be identified and trained, directly after randomization into the intervention group. However, as the Dutch (oncology) physiotherapy community is an expanding and highly motivated group of health care professionals, we expect this procedure to be feasible. Moreover, since this approach strongly resembles the real world situation, results of the study will be well generalizable and, as a consequence of developing a network of trained physiotherapists across the country during the study, an ideal situation is created for future implementation of the exercise program, if proven effective.

Within the field of exercise-oncology, results can be affected by two features. First, effects of the exercise program in the intervention group might be influenced by adherence. Both non-attendance and non-compliance decrease (exercise) treatment dose, thereby resulting in smaller effect sizes. In order to increase attendance and compliance, we implemented several strategies in our study design. In addition to the short distance to the training facilities, these consist of individual guidance by a trained and experienced physiotherapist, an individualized exercise protocol and regular phone calls with the research team. Though attendance and compliance are important, in studies they are rarely reported [39]. In the present study, attendance and compliance to the supervised exercise program will be strictly registered by the physiotherapist and regularly checked by the researchers, while the documented activities in the exercise log of the participants enable us to calculate compliance with the exercise advice.

Second, effects of the exercise program might be influenced by so-called contamination (non-compliance) of the usual care group. This phenomenon is reported to occur in 37% of exercise-oncology trials and is caused by the fact that patients who participate in these type of trials are often highly motivated to exercise, which subsequently leads to an increase of physical activity levels, not only in patients randomized to the intervention group, but also in those randomized to the usual care group [40]. To prevent contamination, we include physically inactive patients (i.e., they might have less natural preference to adapt high physical activity levels when randomized to usual care), elaborate on the randomized design during the informed consent procedure in order to avoid disappointment when being randomized to the usual care group, and repeatedly stress the importance of the usual care group during the inclusion period. After completion of the trial, patients who were randomized to the usual care group can ask the study team for an exercise advice.

In conclusion, the PERFECT study is the first study to investigate effects of an exercise program in esophageal cancer patients after surgery. As the design closely resembles daily practice, results of this study can contribute both to evidence on effects of exercise in esophageal cancer patients, and potential implementation strategies.

## Abbreviations

CPET: Cardiopulmonary exercise testing
EORTC-QLQ-C30: European Organisation for Research and Treatment of Cancer Quality of Life Questionnaire C30
HRQoL: Health-related quality of life
HRR: Heart rate reserve
MFI: Multidimensional Fatigue Inventory
PERFECT: Physical ExeRcise Following Esophageal Cancer Treatment
RM: Repetition maximum
RPM: Revolutions per minute
VO_2peak_: Peak oxygen uptake
